# Healing Efficiency of CNTs-Modified-UF Microcapsules That Provide Higher Electrical Conductivity and EMI Shielding Properties

**DOI:** 10.3390/polym13162753

**Published:** 2021-08-17

**Authors:** Maria Kosarli, Anastasia Polymerou, Georgios Foteinidis, Christos Vazouras, Alkiviadis S. Paipetis

**Affiliations:** 1 Department of Materials Science & Engineering, University of Ioannina, 45110 Ioannina, Greece; m.kosarli@uoi.gr (M.K.); anastpolym@gmail.com (A.P.); g.foteinidis@uoi.gr (G.F.); 2 Telecommunications Laboratory, Sector of Combat Systems, Naval Operations, Electronics and Telecommunications, Hellenic Naval Academy, 18539 Piraeus, Greece; chvazour@hna.gr

**Keywords:** multi-functional materials, self-healing polymers, EMI shielding properties

## Abstract

In this study, the effect of the addition of multi-walled carbon nanotubes (MWCNTs), at three percentages, into the urea-formaldehyde (UF) shell-wall of microcapsules on the healing efficiency is reported. The modified shell-wall created a conductive network in semi-conductive epoxies, which led to an improvement of the electromagnetic interference shielding effectiveness (EMI SE); utilizing the excellent electrical properties of the CNTs. The microcapsule’s mean diameter and shell wall were examined via scanning electron microscopy (SEM). Thermal stability was evaluated via thermogravimetric analysis (TGA). The healing efficiency was assessed in terms of fracture toughness, while the electrical properties were measured using impedance spectroscopy. The measurements of the EMI SE were carried out in the frequency range of 7–9 GHz. The derived results indicated that the incorporation of the CNTs resulted in a decrease in the mean size of the microcapsules, while the thermal stability remained unchanged. In particular, the introduction of 0.5% *w*/*v* CNTs did not affect the healing efficiency, while it increased the initial mechanical properties of the epoxy after the incorporation of the self-healing system by 27%. At the same time, it led to the formation of a conductive network, providing electrical conductivity to the epoxies. The experimental results showed that the SE increased on average 5 dB or more after introducing conductive microcapsules.

## 1. Introduction

Multifunctional composites represent a state-of-the-art technology, since they are capable of simultaneously performing two or more functions [1]. Among their functionalities, such as energy harvesting [2,3,4] and structural health monitoring (SHM) [5,6,7,8], self-healing is being vigorously studied [9,10]. Self-healing can be intrinsic [11,12,13,14] or extrinsic, with the incorporation of vascules [15] or capsules [16] inside the material. Capsule-based composite materials have attracted scientific interest due to their ease of application and mass production, with simultaneously high healing efficiencies [17]. Inside the capsule shell wall lies the healing agent, and upon damage, the shell breaks, and the self-healing process is triggered [18]. There is a variety of healing mechanisms [19], with the most common being the capsule–catalyst system, whereby the healing agent polymerizes after contact with the dispersed catalyst into the polymer matrix [20,21,22]. 

One of the most significant characteristics that capsules should possess is a shell wall strong enough to withstand incorporation in the matrix, but at the same time, thin and brittle enough to break at the locus of damage, to perform successful healing. Of primary importance is that the exterior part of the wall is rough, in order to provide better interlocking with the matrix. In this way, a strong capsule–matrix interface allows the crack to propagate through the capsule, thus delivering the healing agent to the damaged area. Different capsule shell walls have been reported, such as PMMA [23,24,25], polyurethane [26,27], melamine-formaldehyde [28,29,30], and urea-formaldehyde (UF) [31,32,33]. As expected, the mechanical properties of the capsule wall strongly depend on their material [34,35,36]. One material that has been shown to satisfy the above criteria is UF. A UF capsule shell wall provides desirable mechanical properties and easy mass-production via emulsification polymerization. Fereidoon [37] and his co-workers investigated the effect of the addition of nanoparticles (single-walled carbon nanotubes or nano-alumina) to a UF shell-wall on the morphology and the thermal properties of the microcapsules. Ghorbanzadeh Ahangari et al. [38] estimated the effect on the micromechanical and surface properties. The results indicated that the nanoparticles imparted thermal stability, water resistance, a small contact angle, and, most significantly, stiffness and hardness to the shell-wall of the capsules. In contrast, the surface was smoother and the average size reduced, while the core content was not affected. However, there was no subsequent study on the influence on healing efficiency. 

Self-healing is not performed only in terms of mechanical performance but for other properties too. In our recent work [39], we studied the concurrent recovery of mechanical and electrical properties in nano-modified capsule-based self-healing epoxies. Commercial UF microcapsules were modified with the dispersion of MWCTNs into the healing agent, in order to simultaneously restore both the mechanical and electrical properties of the nanomodified, conductive polymer matrix. Several studies were performed for the recovery of electrical properties [40,41,42], thermoelectric properties [43], and electromagnetic interference shielding properties [44,45] of polymer composites. In modern society, electronic systems and telecommunications are the most widely used technologies, as they are essential, integral parts of satellites and aeronautical structures and perform an important role in communication, both on earth and in space. However, electromagnetic interference (EMI) is a significant issue for modern communication and electronic systems [46]. The effects of EMI can range from data loss to system failure and, in extreme cases, loss of life. Traditionally, the shielding of electromagnetic interference is achieved with metal sheets shaped into a suitable form to fit electronic housings [47]. However, due to their high specific weight, traditional conductive metals are now replaced by light conductive materials with high shielding capabilities, such as polymer composites. The reinforcement of polymeric materials with conductive fillers, such as carbon nanofibers (CNFs), carbon nanotubes (CNTs), carbon black, graphene oxide, and graphene, can improve EMI shielding capabilities.

In this study, the UF shell-wall was modified to improve the electrical properties of the polymer matrix in comparison to the conventional microcapsules. Three different percentages (0.3% *w*/*v*, 0.5% *w*/*v,* and 1.0% *w*/*v*) of MWCTNs were dispersed into the EMA solution. The effect of the nanomodification was investigated in terms of capsule properties (mean size and thermal properties) and healing efficiency (fracture toughness tests). The electrical properties and EMI shielding properties were evaluated after incorporating the modified microcapsules into an epoxy matrix and composite material, respectively. 

## 2. Materials and Methods

### 2.1. Materials

For the formation of the shell-wall, urea (NH_2_CONH_2_), formalin (37 wt. % in water), ammonium chloride (NH_4_Cl), resorcinol (C_6_H_4_-1,3-(OH)_2_), and poly(ethylene-maleic-anhydride) (EMA, M_w_ = 100,000–500,000 g/mol) copolymer powder were supplied from Sigma-Aldrich (Athens, Greece). Diglycidyl ether of bisphenol-A (DGEBA, Epikote 828 lvel) epoxy resin and Epikure 541 hardener, supplied by Dichem Polymers, Greece, were selected as the healing agent and matrix phase, respectively. The non-toxic solvent ethyl-phenylacetate (EPA) was used to decrease the viscosity of the resin. The nanomodification of the shell-wall and the matrix was performed using Graphistrength C-100Multi-Wall Carbon Nanotubes (MWCNTs) from ARKEMA, France. The length and diameter of the MWCNTs ranged from 1 to 10 μm and 10 to 15 nm, respectively. As a catalyst for the healing process, the Aluminium(III) triflate (Al(OTf)_3_) was selected.

### 2.2. Nanomodification of the Shell-Wall

A 2.5% *w*/*v* aqueous solution was prepared by adding 2.5 g EMA powder to 100 mL of deionized water in a beaker and mixed overnight in a warm bath. MWCNTs were dispersed in the surfactant at 3 different percentages (0.3% *w*/*v*, 0.5% *w*/*v,* and 1% *w*/*v*) by sonication using a UP400S by Hielscher SA, Germany. The amplitude was 0.3 and the pulse 0.5. The sonication duration was 90 min, while every 30 min the process was paused, and the mixture was magnetically stirred for 5 min. At the end of the process, the solution was filtrated to remove the agglomerations of CNTs that were not distributed in the surfactant.

### 2.3. Encapsulation Process

Initially, 25 mL of the surfactant solution were added to a high shear mixer (Dispermat AE, VMA-GETZMANN DMBH, Reichshof, Germany, Getzmann) with 100 mL of deionized water under continuous stirring at 500 rpm in each case. Then, 2.5 g urea, 0.25 g resorcinol, and 0.25 g ammonium chloride were placed in the beaker. When the solution became clear, 60 g of resin/solvent with a 5 wt. % dilution was added. After 15 min, 6.33 g formalin was placed in the mixer, the temperature was increased to 55 °C at a rate of 10 °C/min, and the reaction was left to proceed for 2 h. After the encapsulation process, the mixture was cooled down to room temperature, the suspended microcapsules were rinsed with ethanol using a Buchner filter, and finally were left to dry. Hereafter, the produced capsules will be referred to as “neat capsules” for the unmodified (neat) capsules and “0.3% capsules”, “0.5% capsules”, and “1% capsules” for the capsules with a nanomodified wall containing 0.3% *w*/*v*, 0.5% *w*/*v,* and 1% *w*/*v* CNTs, respectively. 

### 2.4. Manufacturing of the Self-Healing Specimens

Healing efficiency was evaluated in terms of fracture toughness using modified tapered double cantilever beam geometry [48]. In this case, the central groove was the self-healing section that included the microcapsules and the catalyst for the evaluation of the healing efficiency. A two-part nanomodified with 0.3 wt. % MWCNT epoxy system (Epikote 828-Epikure 541) was used as matrix phase at a 100:50 ratio. The microcapsules and the catalyst were dispersed at 20 wt. % and 1.5 wt. % in the matrix, respectively, and the mixture was mechanically stirred for 5 min. As was already reported, [39] the curing reaction of these metal triflates is based on Lewis acid-catalyzed chemistry and ring-opening polymerization. The healing process starts when the encapsulated healing agent is released from the capsules and encounters the catalyst. This process does not affect the incorporation of MWCNT’s in the polymeric shell-wall, since the wall is destroyed after breakage of the capsule. At the end of this process, the mixture was placed in a vacuum laboratory oven for degassing for 2 min and cast in silicon molds. The curing process took place at room temperature for 24 h. In order to estimate the effect of the incorporation of the self-healing system on the initial mechanical properties, specimens without capsules and catalyst were manufactured as “reference” samples. Hereafter, TDCB specimens with neat capsules will be referred to as “neat system”, and specimens with 0.3%, 0.5%, and 1% capsules will be called “0.3% system”, “0.5% system”, and “1% system”, respectively. All specimens were pre-cracked using a fresh razor blade and tested (virgin specimens) using a mini tensile testing frame manufactured by Fullam Inc. USA, with a 1 mm/min displacement rate. The healing process took place at 60 °C for 24 h in a laboratory oven. The specimens were retested after healing using the same experimental conditions (healed specimens). The alteration of the initial mechanical properties was evaluated using Equation (1). The healing efficiency was calculated as the ratio of the peak load of the virgin specimen to the peak load of the healed specimen, Equation (2).

(1) $$k\%=1-\frac{P_{c}^{Virgin}}{P_{c}^{Reference}}\times 100\%$$

(2) $$n\%=\frac{P_{c}^{Healed}}{P_{c}^{Virgin}}\times 100\%$$

### 2.5. Manufacturing of the Specimens for Electrical Conductivity Measurements

Impedance spectroscopy was employed to investigate the effect of the introduction of the microcapsules in the nano-enhanced matrix. A low CNT loading was also employed to modify the epoxy matrix to impart the necessary conductivity for reasonably fast impedance measurements after the incorporation of the capsules inside the network. The 0.3 wt. % MWCNTs were dispersed in Diglycidyl ether of bisphenol-A (DGEBA, Epikote 828 lvel) epoxy resin, based on a previous study [3]. This study indicated that the percolation threshold was reached at 0.5 wt. % MWCNTs. In this study, a 0.3 wt. % CNT loading was selected to investigate the effect of introducing different types of capsules on the electrical properties. This percentage was chosen, as it is close to the aforementioned percolation threshold. As was observed, the chosen 0.3% CNT loading, in conjunction with the introduction of nanomodified capsules, led to an electrically percolated system. The dispersion process lasted 3 h in the dissolver at 3000 rpm and at room temperature. After the dispersion process, the microcapsules were introduced into the nanomodified resin, and the system was mixed with Epikure 541 hardener in a 100:50 ratio. The mixture was finally cast in silicon rubber molds. The specimens for electrical characterization had dimensions of 52 × 12 × 3 mm^3^.

### 2.6. Manufacturing of the Specimens for EMI Shielding Measurements

For the evaluation of the EMI shielding properties, before and after the introduction of the self-healing system, carbon fiber reinforced polymer composite panels with a nano-modified epoxy matrix were fabricated via a hand lay-up technique using vacuum bagging. The nano-modified epoxy matrix (DGEBA, Epikote 828) was employed for both systems. First, 20 wt% of the 0.5% nanomodified capsules were introduced into the matrix, together with 1.5 wt% catalyst. The 0.5% nanomodified capsules were chosen as they exhibited a combination of satisfactory healing efficiency and increased initial mechanical properties, together with electrical percolation. The capsule and catalyst percentages were chosen as they have been reported to impart the optimal properties to the self-healing matrix [4]. Finally, UD (unidirectional) glass fiber reinforced laminates were manufactured by hand lay-up. The size of the panels was 500 × 500 mm^2^.

### 2.7. Characterization Techniques

#### 2.7.1. Scanning Electron Microscopy (SEM)

The mean diameter of 100 individual capsules was evaluated via SEM images. The morphological characterization was performed using a JEOL JSM 6510LV, Oxford Instruments (Abingdon, UK), scanning electron microscope. The microcapsules were gold-platinum sputter coated to avoid their decomposition due to the high applied voltage, set at 5 kV.

#### 2.7.2. Thermogravimetric Analysis (TGA) 

The thermal stability of the microcapsules was examined via thermogravimetric analysis (TGA). TGA scans were performed between 25 °C and 700 °C, with a heating rate of 10 °C/min, under Ar atmosphere. The mass of each sample was 3.8 mg and the gas flow 60 mL/min. The measurements were performed using a PerkinElmer Pyris Diamond TG/DTA instrument. 

#### 2.7.3. Impedance Spectroscopy

An advanced dielectric thermal analysis system (DETA-SCOPE), supplied by ADVISE Greece, was utilized for the impedance measurements. The specimens were placed inside a capacitor, consisting of two copper plates with dimensions of 52 mm × 12 mm. A sinusoidal excitation of 10 V was applied between the capacitor’s electrodes. Impedance spectra ranged from 0.01 Hz to 100 kHz. All measurements were performed under a stable temperature of 25 ± 0.1 °C, continuously measured using an EXTECH VIR50 IR thermometer. 

#### 2.7.4. EMI Shielding

The shielding efficiency of the specimens was measured utilizing a Keysight P3972A 300 kHz–9 GHz vector network analyzer (VNA). Measurements were carried out in the microwave frequency region of 7–9 GHz using a pair of identical rectangular horn antennas for transmission and reception. The rectangular horn aperture dimensions were 7.5 × 7.5 cm^2^, with an E plane slant height of 16.48 cm and an H plane slant height of 20.64 cm, corresponding to a gain of approximately 16.7 dBi at 9 GHz. The antennas were placed at a distance of approximately 1.5 m. The 500 × 500 mm^2^ rectangular composite laminates were placed in the middle between the two antennas at normal incidence. Thus, the laminates were in the far-field region of both antennas, while fully blocking the first few (at least 4) Fresnel zones throughout the frequency region tested. This setup may be expected to approximate the overall shielding efficiency for relatively large composite sheets, while in practice averaging over possible spatial differences in the structure across the sheet that might have arisen due to the fabrication process. At the same time it allows for minimal specimen preparation and easy replacement.

The shielding effectiveness (SE) for a specimen is defined as

$$\left(SE\right)=10log{\left(\frac{P_{i}}{P_{o}}\right)}_{S}-10log{\left(\frac{P_{i}}{P_{o}}\right)}_{V}={\left({\left|S_{21}\right|}^{2}\right)}_{dB}{}^{S}-{\left({\left|S_{21}\right|}^{2}\right)}_{dB}{}^{V}$$

where “S” stands for “specimen” and “V” for “void”, i.e., the difference in power transmission coefficient caused by the presence of the specimen. Thus, there was no need to “de-embed” the specimen; i.e., account for additional losses along the signal path between the two ports of the VNA, since they are canceled out by subtraction. Since our primary focus was on the exploration of improvement in SE by changes in the composite structure, we could calculate the shielding effectiveness improvement (SEI) between materials with and without the capsules under consideration; the first one taken as a modified material, which is the specimen with capsules, and the second one taken as a reference material, which is the specimen without capsules.

$$\left(SEI\right)={\left(SE\right)}_{modified}-{\left(SE\right)}_{reference}={\left({\left|S_{21}\right|}^{2}\right)}_{dB}{}^{modified}-{\left({\left|S_{21}\right|}^{2}\right)}_{dB}{}^{reference}$$

## 3. Results and Discussion

### 3.1. Size Distribution/SEM Images

**Figure 1:**
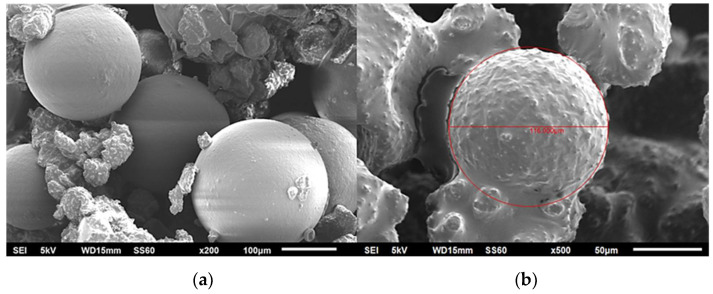
SEM images of (**a**) unmodified (neat) capsules and (**b**) capsules with nanomodified shell-wall at 0.5% *w*/*v*.

**Figure 2:**
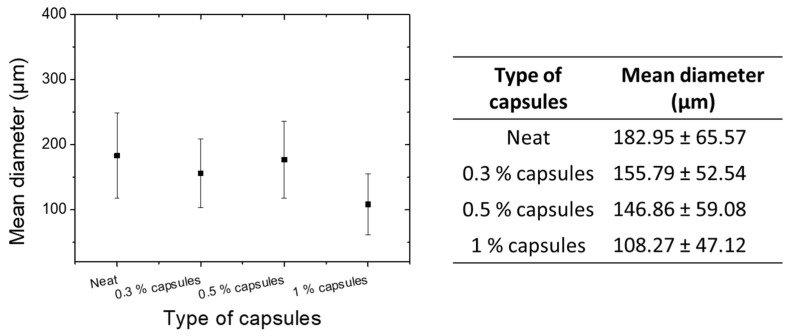
Mean diameter of the different capsule types.

Figure 1a,b illustrates SEM images of the neat capsules and those with a CNT modified shell wall, respectively. As observed, the shell wall in the case of the unmodified capsules was smooth. This was due to the shorter duration of the encapsulation process, since the desired roughness of the wall was observed after the second hour of polymerization [5]. For the modified capsules, the shell-wall was rough and thicker, due to the CNT incorporation. The results indicated that the mean size decreased after the addition of the CNTs in the shell wall (Figure 2). As was also observed in the literature [6,7], smaller microcapsules were produced after the incorporation of nanoparticles, due to the reduced number of collisions between the smaller droplets. The outer UF shell of the microcapsules with a rougher surface were thicker than those of the microcapsules with smoother surfaces. The surface roughness of the microcapsules increased upon addition of the nanoparticles, which was most likely due to the increase in the condensation rate of the PUF particles in the modified capsules.

### 3.2. Thermal Stability of Microcapsules

**Figure 3:**
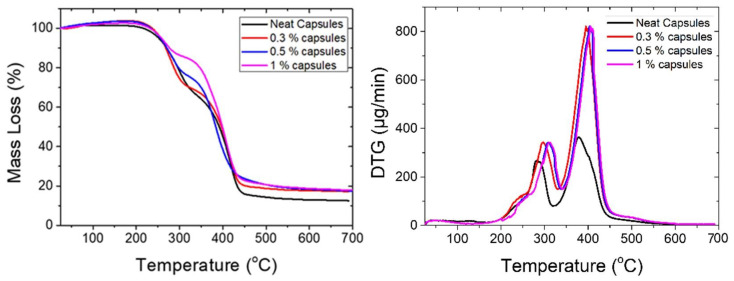
TGA measurements (**left**) and DTG measurements (**right**) of neat capsules (black line), 0.3% capsules (red line), 0.5% capsules (blue line), and 1% capsules (purple line).

The thermal stability of the microcapsules was investigated via thermogravimetric analysis upon heating from 25 °C to 800 °C, with a scanning rate of 10 °C/min in a nitrogen atmosphere (Figure 3, left). Capsules were allowed to dry at 60 °C for 2 h before the scans, in order to remove any residual water. The mass loss was manifested in two main steps for all capsule batches. More specifically, the first step of the mass loss was observed at a temperature range from ca. 250 °C to 360 °C for the neat capsules, while for the 0.3%, 0.5%, and 1% capsules, the ranges were 264 °C to 330 °C, 240 °C to 340 °C, and 240 °C to 330 °C, respectively. This first mass loss was associated with the thermal decomposition of the poly(UF) shell wall material and the starting point of the evaporation of the solvent. The above measured values of minimum temperature for each range define the maximum temperatures at which the capsules can be used. The second step was observed at a temperature range from ca. 360 °C to 440 °C for the neat capsules and from ca. 340 °C to 440 °C, 370 °C to 440 °C, and 350 °C to 420 °C for the 0.3%, 0.5%, and 1% capsules, respectively. The second step of the mass loss was attributed to homopolymerization of the encapsulated resin. During continuous heating at high temperatures, the encapsulated epoxy resin is partially polymerized, with the onset of polymerization observed at 450 °C. The obtained results clearly indicated that the capsules remained thermally stable at high temperatures (approximately up to 230 °C). All the aforementioned steps of mass loss and the maximum temperatures that the capsules can be used were confirmed via DTG measurements (Figure 3, right).

### 3.3. Evaluation of the Healing Efficiency

**Figure 4:**
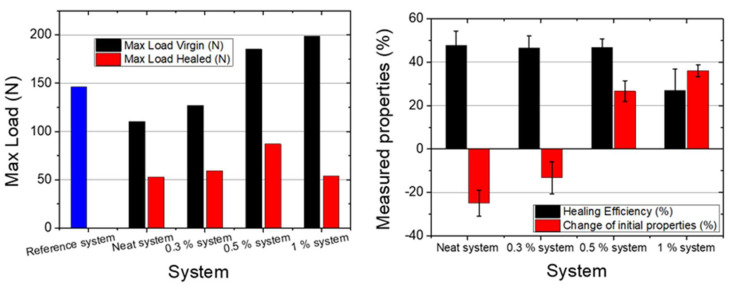
Bar charts of maximum load of virgin and healed specimens (**left**) and of healing efficiency and change of initial properties (**right**) of all capsule systems.

The healing efficiency was evaluated in terms of fracture toughness (Figure 4). TDCB specimens with neat microcapsules (neat system) exhibited a healing efficiency of about 48%, while the 0.3% system and 0.5% system provided the same values (47%) (Table 1). However, in the case of the 1% system, the healing efficiency was significantly reduced, and revealed values of about 27%. This can be attributed to the increased strength of the UF shell-wall due to the incorporation of a greater quantity of CNTs. As is well-known, the incorporation of CNTs enhances both the mechanical and electrical properties in polymeric materials [15,49,50,51,52,53], making the rupture of the shell wall more difficult. 

The effect on the initial mechanical properties of the epoxy matrix after the introduction of the self-healing system was also evaluated. A substantial decrease, of ca. −25%, in the peak load was observed in the neat systems. Capsules were easily detached from the matrix due to their smooth external surface, which led to the deterioration of the fracture toughness and subsequently this significant decrease. This was not observed when the 0.3% CNTs were introduced into the capsule shell walls, where the reduction in strength was ca. −13%. This was attributed to the fact that the incorporation of CNTs into the polymer shell wall improved the interlocking with the matrix due to the increased roughness of the exterior wall, as shown in Figure 1. Additionally, for the 0.5% system, the mechanical properties increased after the incorporation of the self-healing agent, by ca. +26%. As the percentage of CNTs was increased, the mechanical strength of the shell wall was also increased, and this subsequently led to improved properties. This was also observed in the case of the 1% system. However, since in this case the healing efficiency was decreased, this system was not identical. The optimal choice seemed to be the 0.5% system, since it had increased initial mechanical properties and a similar healing efficiency to the neat system.

### 3.4. Conductive Network

**Figure 5:**
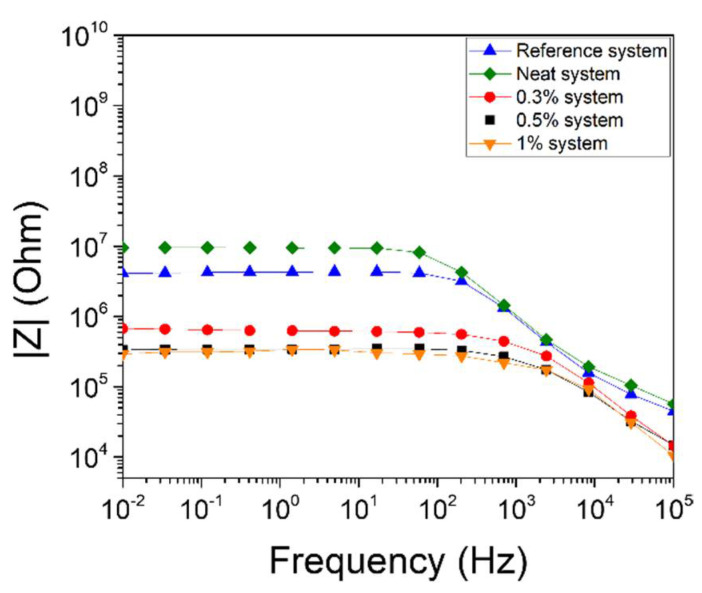
Bode plots of impedance measurements.

Impedance spectroscopy was utilized in order to examine the effect of the incorporation of the unmodified and nanomodified microcapsules into the nano-enhanced composite matrix. Figure 5 shows Bode plots of the magnitude of the impedance versus frequency. IS scans were made for five different types of specimen: (i) the reference specimens (without microcapsules), and the specimens with incorporated (ii) neat capsules, (iii) 0.3% capsules, (iv) 0.5% capsules, and (v) 1% capsules.

The specimens with neat capsules (green curve) exhibited an increase in the magnitude of their impedance at low frequencies, where the material had an Ohmic behavior (frequency independent), in comparison with the reference specimens (blue curve). Additionally, the transition of the Ohmic to non-Ohmic behavior shifted to lower frequencies. The above observations indicate that the inclusion of neat microcapsules reduces the electrical conductivity of the matrix by interfering inside the conductive CNT network. More specifically, the impedance increased due to reduction of the conductive paths in the CNT network, as the non-conductive microcapsules acted as current barriers inside the nanocomposite.

On the contrary, the nano-enhanced microcapsules improved the electrical conductivity of the nanocomposite, even after the incorporation of small loadings. In the case of the introduction of 0.3% capsules, the IS scan (red curve) showed a significant decrease in the magnitude of the impedance compared with the reference specimen. The Ohmic part of the curve had a drop from 3.76 × 10^6^ Ohm to almost 6.75 × 10^5^ Ohm, and the Ohmic to non-Ohmic behavior shifted from 200 Hz to 2 kHz. A further increase of the CNT loading in the shell wall led to even more conductive systems. The incorporation of 0.5% capsules (black curve) exhibited a more conductive behavior, while the most conductive system was observed when 1.0% capsules were introduced (orange curve). The impedance values of these specimens reached the values of the percolated system (at 0.5 wt. %), as measured in a previous study [49]. A decrease of more than one order of magnitude, compared to the specimen with unmodified capsules, was attained in all cases where CNT modified capsules were employed and ca. one order of magnitude in the case where no capsules were introduced into the matrix. This observation indicates that the conductive microcapsules operate synergistically with the CNT network and further contribute to the improvement of the conductivity of the matrix.

### 3.5. EMI Shielding Properties

**Figure 6:**
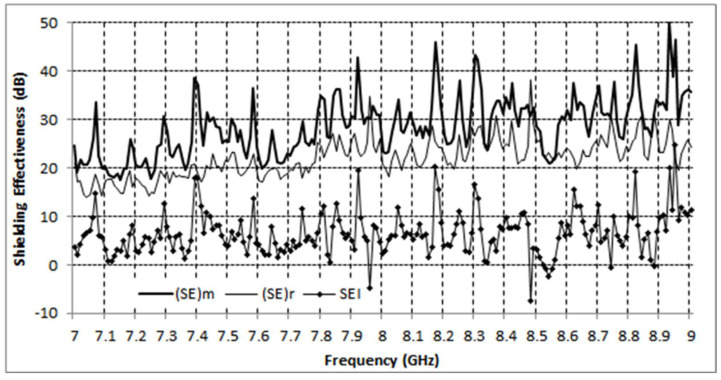
Shielding effectiveness for the modified specimen and the reference specimen. ${\left(\mathrm{SE}\right)}_{m}$ and ${\left(\mathrm{SE}\right)}_{r}$ stand for ${\left(\mathrm{SE}\right)}_{\mathrm{modified}}$ and ${\left(\mathrm{SE}\right)}_{\mathrm{reference}}$, respectively, and (SEI) denotes the shielding effectiveness improvement.

The results for the capsule specimens, as compared with the reference specimen, are shown in Figure 6.

A consistent increase in shielding effectiveness of around 5 dB between the two materials may be observed, indicating a clear improvement over the reference material, with a minor exception only in the vicinity of 8.5 GHz. The fluctuation pattern seen in Figure 6 can be attributed to multiple reflections along the signal paths between the VNA ports and the specimen. A similar pattern was exhibited by the modified material, as well as the reference material, being more pronounced in the former; this indicates a stronger reflection from the modified specimen, consistent with an increase in conductivity due to the incorporation of microcapsules. Overall, the shielding effectiveness is quite high for both specimens, as has already been observed in previous studies (see e.g., [54,55,56] and references therein) on CNT based nanocomposite materials.

Some preliminary measurements [55] were also carried out for the radar cross section (RCS) of the specimens, which is closely related to the surface power reflection coefficient R for the material under consideration. More specifically, according to a well-known result (e.g., [56] Sections 7.4.3, 7.5.1.3) based on physical optics for lossy dielectric strips or discs which are very large compared to the wavelength, the backscatter cross section σ for normal incidence is given by

$$\sigma =R\;\sigma_{pc}$$

where $\sigma_{pc}$ is the backscatter cross section of a perfectly conducting plate of the same dimensions, which in turn is easily calculated by physical optics. Our measurements [55] yielded a backscatter cross section more than 10 dB below the standard physical optics result $\sigma_{pc}$ for the perfectly conducting plate, corresponding to a value of R well below 0.1. Hence, the behavior of the materials tested appeared to be in the region of low reflected power demonstrated in [54], corresponding to a relatively low nanofiller content, while absorption clearly seemed the dominant shielding mechanism. A more detailed investigation of the EMI shielding properties of the materials under consideration is envisaged for a future study.

## 4. Conclusions

In this study, electrically conductive microcapsules were produced via the incorporation of multi-wall carbon nanotubes into polymer urea-formaldehyde shell-walls. An in situ emulsification polymerization was used as the encapsulation technique. Neat (commercial) capsules and capsules with three different percentages (0.3% *w*/*v*, 0.5% *w*/*v,* and 1% *w*/*v*) of carbon nanotubes in their shell-wall were manufactured. The mean diameter of the capsules was found to decrease as the nanofiller percentage increased. In addition, the roughness of the exterior wall was substantially improved. The modification did not alter the thermal stability of the polymeric shell. 

Microcapsules were incorporated in a nanomodified DGEBA polymer matrix to evaluate the effect of the modification of the wall on the healing efficiency and on the change in the initial mechanical properties after incorporating the self-healing system. TDCB fracture toughness tests of the nanomodified resin with capsules with an increasing percentage of CNTs also exhibited improvement in the initial mechanical properties. More specifically, the incorporation of neat capsules into the matrix showed a reduction of the mechanical properties of about −25% compared to the neat matrix. On the other hand, the incorporation of 1% capsules led to an increase of 36% in the fracture toughness. The healing efficiency was measured at ca. 47% and did not change with the introduction of 0.3% and 0.5% capsules. However, in the case of the 1% capsules it decreased ca. 20%. This can be attributed to the limited capsule breakage, as the addition of CNTs into the capsule wall significantly increased their mechanical strength, and the capsules did not break and deliver the healing agent. 

The effect of the modification of the capsule’s shell wall after their incorporation in a modified polymer matrix was measured in terms of electrical properties using the impedance spectroscopy technique. The polymer matrix was modified with 0.3 wt.% MWCNTs. As aforementioned, this CNT loading is below the percolation threshold. However, the addition of modified capsules resulted in a decrease in the magnitude of the impedance, to values indicating that percolation was achieved via combined CNT and microcapsule loading. 

Finally, both the reference (without capsules) and self-healing systems (with capsules) were measured in terms of their EMI SE. The self-healing system was then added to carbon reinforced composites with the same nanomodified polymer matrix, to evaluate the change of the shielding properties. A consistent increase in shielding effectiveness, of around 5 dB, between the two materials was assessed, indicating a clear improvement over the reference material, with a minor exception only in the vicinity of 8.5 GHz. 

## Figures and Tables

**Table 1:** Healing efficiency and change of initial mechanical properties of all categories.

System	Max Load Virgin (N)	Max Load Healed (N)	Healing Efficiency (%)	Change of Initial Properties (%)
Reference system	146.2	-	-	-
Neat system	110.2	52.7	47.8 ± 6.6	−24.9 ± 6.0
0.3% system	126.9	59.2	46.6 ± 5.5	−13.2 ± 7.4
0.5% system	185.2	87.0	46.9 ± 3.8	+26.7 ± 4.8
1% system	198.9	53.7	27.0 ± 9.9	+36.1 ± 2.7
